# Effect of on-table extubation after congenital heart surgery on outcomes in a developing country

**DOI:** 10.1186/1749-8090-10-S1-A281

**Published:** 2015-12-16

**Authors:** Zeena Makhija, Amit Kumar, Mahesh Bhatt

**Affiliations:** 1 Department of Congenital Cardiothoracic surgery, Shree Krishna Hospital, Gujarat, India; 2 Department of Pediatric Cardiac Intensive Care, Shree Krishna Hospital, Gujarat, India; 3 Department of Pediatric Cardiology, Shree Krishna Hospital, Gujarat, India

## Background/Introduction

Recent advances in anaesthesiology and critical care have made it feasible to extubate children on-table after surgical repair of congenital heart defects.

## Aims/Objectives

We compared two propensity matched groups to evaluate the effect of on-table extubation on outcomes.

## Method

144 patients underwent surgical repair for various congenital heart defects in our institution between February 2014 and April 2015. 34 patients who were extubated on table (group A) were compared with propensity matched group of 34 patients who were extubated later in the ICU(group B). Re-intubation, significant bleeding, low cardiac output syndrome, arrhythmia in PICU, ICU stay, hospital stay and hospital cost were analysed.

## Results

Demographics of both the groups were similar. Mean age at time of operation was 4.1 ± 3.8 years. Seventy-nine percent (n = 27) were <5 years old and 70% (n = 24) were males. Ventricular septal defect (38%, n = 13) was the most common lesion, followed by atrial septal defect (35%, n = 12) which were repaired. Cardiopulmonary bypass and aortic cross clamp times were 65.3 ± 32.2 and 31.3 ± 22.8 minutes, respectively. The mean inotrope score was 2.1 ± 1.3. There was no mortality in the cohort and no complications during PICU stay. None of the patients required re-intubation. The mean length of PICU stay was 1.4 ± 0.8 days in group A which was not statistically significantly different from group B (1.8 ± 0.6 days) (p value: 0.6). The total hospital stay and the hospital cost were also similar in both groups.

## Discussion/Conclusion

On table extubation is safe and feasible in a selected group of patients who undergo congenital heart surgery. It lessens the duration of ICU stay, total hospital stay and hospital expenditure.

